# Hemangiopericytoma: Conducts and perioperative management of an extent sinonasal tumor in a Jehovah's Witnesses patient ‐ Case report

**DOI:** 10.1002/cnr2.1609

**Published:** 2022-02-23

**Authors:** Francisco S. Amorim Filho, Flávio M. Gripp, Guilherme S. Faria, Mateus Capuzzo Gonçalves, Lincoln Miyahira

**Affiliations:** ^1^ Head and Neck Surgery and Otorhinolaryngology Service Instituto da Tireoide e Laringe Goiânia Brazil; ^2^ Department of Otorhinolaryngology and Head and Neck Surgery Hospital das Clínicas de Goiânia (HC‐UFG) Goiânia Brazil; ^3^ Department of Otorhinolaryngology and Head and Neck Surgery University of Campinas (UNICAMP) Campinas Brazil

**Keywords:** head and neck neoplasms, hemangiopericytoma, Jehovah's Witnesses, perioperative care, transanal endoscopic surgery

## Abstract

**Background:**

Hemangiopericytomas (HPCs) are rare tumors derived from mesenchymal cells with pericyte differentiation. About 5% of head and neck HPCs occur in the nasal cavity and paranasal sinuses. Due to its rarity, rich vascularity and variable biological behavior, its management is a challenge in itself.

**Case:**

We report a case of sinonasal HPC in a Jehovah's Witness patient and discuss the obstacles and care related to the restrictions and therapeutic challenges involved in the approach to the patient. The patient was successfully treated by endoscopic endonasal approach with all per‐operative care and restrictions being respected and attended.

**Conclusions:**

The management of HPC by itself involves challenges and when associated with other restrictive conditions attention and care are required.

## 1 BACKGROUND

Hemangiopericytomas (HPCs) are rare tumors derived from mesenchymal cells with pericyte differentiation, originated from Zimmermann's pericytes, that forms the outer layer of a normal capillary, in other words, cells that involve the capillary. They have smooth muscle characteristics, being responsible for regulating the caliber of the vessels due to their contractile capacity, and thus, modulate the flow and vascular permeability.^1^, ^2^ This vascular neoplasm has been known by pathologists and surgeons for more than 70 years and was first described in 1942 by Stout and Murray.^3^ Most of the HPCs are histologically benign, but a small percentage have atypical characteristics with uncertain malignancy potential, with some case series demonstrating the presence of metastatic disease in about 12% of cases.^4^

The HPC can affect different anatomic sites of the body, involving more frequently the lower limbs, pelvis and the retroperitoneum.^5^ The head and neck is the third most affected site, so that about 15%–30% of all HPCs are seen in this segment, and of those, only 5% occurs in the nasal cavity and paranasal sinuses, affecting mainly adults in the fifth and sixth decades of life.^1^, ^6^, ^7^ Its etiology is still unknown, although it has been associated by some authors to hypertension, trauma, prolonged use of steroids and hormonal imbalance, exposure to chemicals and radioactive compounds, but such correlations have not yet been demonstrated.^1^, ^2^, ^6^, ^8^, ^9^

As a possible and rare paraneoplastic syndrome likely to occur, there is the tumor‐induced osteomalacia (TIO), characterized by the secretion of fibroblast growth factor 23 (FGF23) mainly by benign mesenchymal tumors and sometimes by malignancies. Patients with osteomalacia complain of progressive bone pain, muscle weakness, walking disability, and other symptoms.^10^

Correctly diagnosing the HPC is not an easy task, however it is essential for an efficient treatment. Because of its rareness and unpredictable biological behavior, this infirmity may cause confusion and uncertainty regarding its treatment and is a significant problem not only for pathologists, but also for surgeons who conduct the case. A long‐term follow‐up of the patient is essential to predict the behavior of the tumor.^6^

However, due to its rare occurrence and unpredictable biological behavior, this disease generally triggers confusion and uncertainty regarding its treatment and conduct.

The purpose of this article is to report a case of sinonasal hemangiopericytoma (SNHPC) in a Jehovah's Witness who was treated with preoperative embolization and endoscopic resection. Our aim is to add our experience to the growing body of literature on this disease, as there are a few cases reported in the literature and to report the first reported case of SNHPC in a patient who could not receive a blood transfusion due to religious issues and to discuss the challenges involving management in this situation and the need of a multidisciplinary approach.

## 2 CASE

A total of 68 years old female was referred to the Head and Neck surgery specialist evaluation after intense epistaxis. Three years ago, she started to present nasal obstruction, more pronounced on the right nostril, associated with sporadic episodes of epistaxis, which became more frequent and intense over the subsequent months. She reported having already undergone previous cauterizations in other hospitals to stop the bleeding. The patient had systemic arterial hypertension (use of amlodipine and losartan), diabetes (use of metformin and glibenclamide) and coronary disease with necessity of three stens (last placement over a year ago). Besides that, there was the fact that the patient refuses treatments that could use blood components by religious belief.

For the Jehovah's Witnesses, the violation of this moral principle means disobeyment and disloyalty to its creator and giver of life. Their faith represents a challenge for the surgeons, who must have basic knowledge and be comprehensive of their doctrines to minimize and control the loss of blood in these patients.^11^

Nasal endoscopy was performed and revealed a gray‐pink to red, soft, hemorrhagic mass that occupied the entire right nasal cavity without identifying the lesion's pedicle that impeded the progression of the endoscope.

**FIGURE 1:**
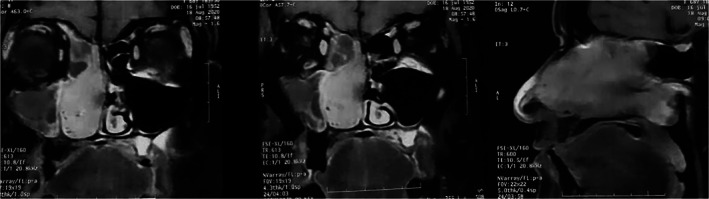
Nuclear magnetic resonance ‐ Sagittal and coronal planes evidencing an extensive tumor in the nasal cavity extending to the right rhinopharynx with important contrast enhancement

To progress the investigation, a NMR of the face was performed and showed an expansive formation with a polypoid aspect occupying the entire length of the right nasal cavity, measuring 8.6 cm. The lesion caused an enlargement of the ipsilateral nasal cavity, without bone erosion or soft tissue infiltration, medially bulging the nasal septum and laterally repelling the right nasal turbinates. Posteriorly, it insinuates itself in the nasopharynx, reducing its light. (Figure 1).

**FIGURE 2:**
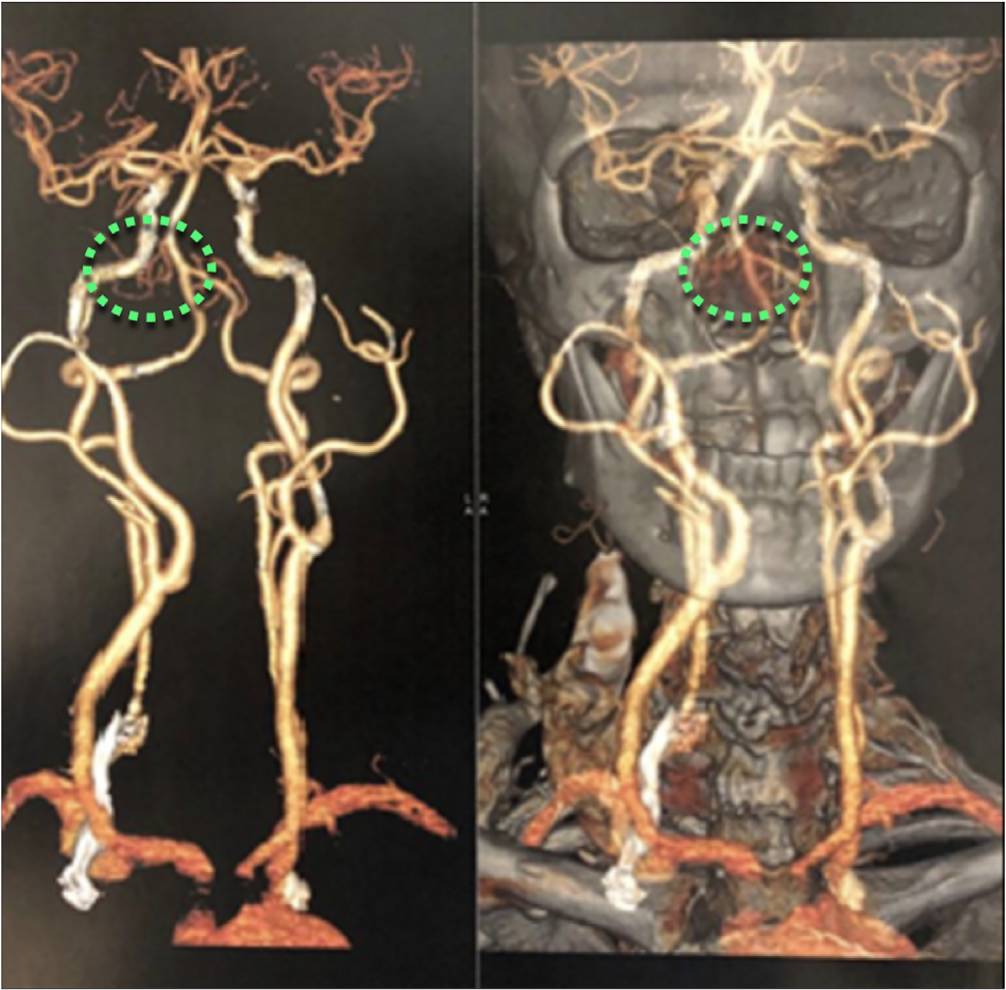
Cervicofacial angiotomography. Doted area: hypervascular lesion, irrigated by branches of the right maxillary artery

An angiotomography was made, revealing an hypervascular lesion, irrigated by branches of the right maxillary artery. There were no major branches of the ophthalmic artery feeding the lesion. (Figure 2).

As the diagnostic hypothesis was a vascularized sinonasal tumor, a surgical approach was planned. The surgery without the possibility of use of blood components required a thorough preoperative planning from the entire team, including a detailed clinical history (hemorrhagic disorders, previous surgery, medications that could affect clotting) and evaluation of exams with the intent to identify coagulation abnormalities and pre‐existing anemia.

In view of the high risk of intraoperative bleeding due to the hypervascular lesion, and the patient's religious belief, it was opted for embolization of the tumor nourishing artery 2 days before surgery, aiming the complete resection of the tumor with safety.

**FIGURE 3:**
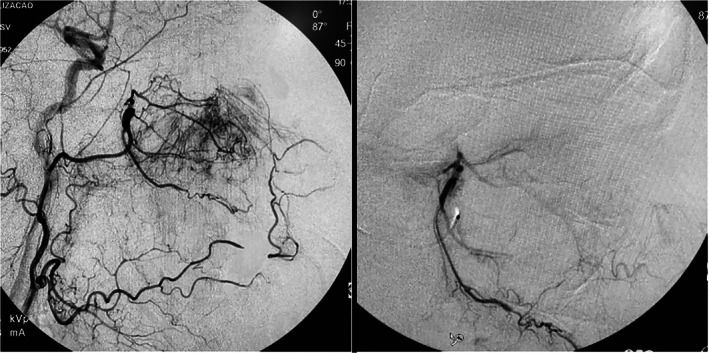
(A) Pre‐embolization arteriography; (B) Post‐embolization arteriography demonstrating interruption in contrast flow

Embolization was performed by catheterization of the right common femoral artery, with angiographic series made with contrast in the following arteries: right internal and external carotid arteries and right internal maxillary artery. The lesion had afferent branches mainly from the right sphenopalatine artery, but it also had ones from ethmoidal arteries, right ophthalmic artery and left sphenopalatine artery. The embolization of the distal branches of both sphenopalatine arteries was selectively performed with 300‐500u PVA microparticles until the complete exclusion of tumor enhancement by iodinated contrast. (Figure 3).

The surgery began by endoscopic approach (transnasal), although the patient was aware that external access (lateral rhinotomy) could be necessary if the first attempt was not enough to completely remove the tumor.

During surgery we faced a reduced size lesion compared to the one sight on imaging exams (reflex of embolization), with precise limits and little local bleeding, easily controlled by cauterization. The tumor was completely removed by endoscopy, and after reviewing the hemostasis, an absorbable hemostatic was placed on the surgical bed and a nasal plug was put on the nose. The patient stand under clinical care, maintaining blood pressure levels, cardiac function and blood glucose within the normal range. Both the nursing and medical team maintained intensive monitoring to identify and quantify any bleeding.

The entire surgical team remained on standby to intervene in the event of postoperative bleeding. The patient remained stable throughout the hospital stay, with the nasal plug up to the 7th postoperative day, without presenting epistaxis, being safely discharged.

The tumor was sent in formaldehyde to the pathology service, being included in paraffin blocks. Measuring 7.5 × 6.8 × 2.0 cm^3^, the lesion was represented by mesenchimal proliferation consisting of polygonal cells, arranged in solid sheets, expansive. The neoplastic cells were isomorphic, free of atypia, containing areas of myxoid degeneration of the matrix. Vascularization was exuberant with endothelial cells flattened by a large number of capillaries with perivascular hyalinization, as well as irregular “deer antler” branching, a hemangiopericytoid pattern.

**FIGURE 4:**
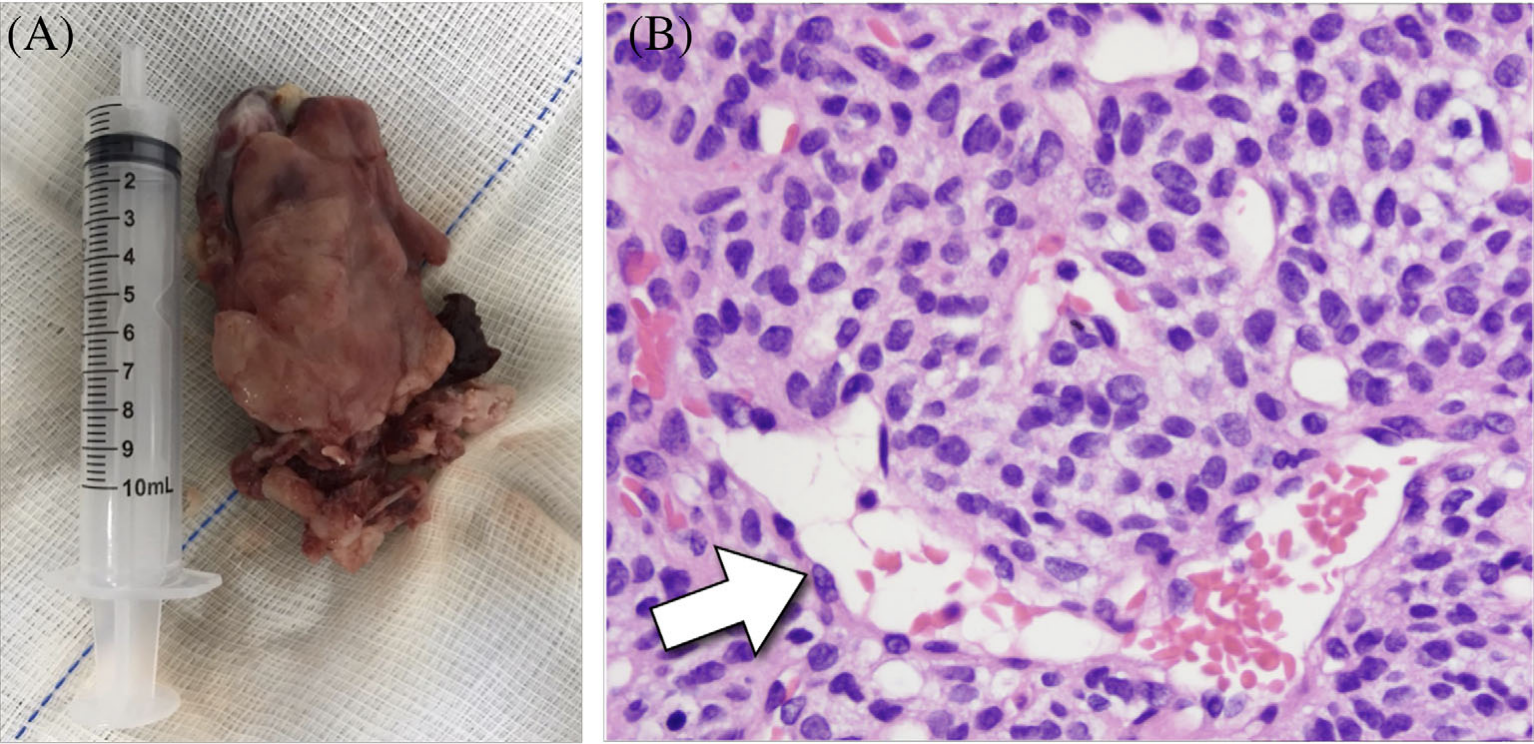
(A) Right nasal cavity tumor; (B) Hemangiopericytoma. White arrow: Vessels with hemangiopericytoid pattern – “deer's antler”

On immunohistochemistry test (IHC), the tumor cells were positive for vimentin, CD 34 and Ki67 limited to the endothelium. Based on these finding, the diagnosis of HPC could be made (Figure 4).

Currently, the patient is in the twelfth month after surgery and the follow‐up shows no signs of recurrence on nasoendoscopy and facial MRI.

The SNHPC evolves painlessly during its growth and progression, and its main symptoms are epistaxis and nasal obstruction. On otorhinolaryngological examination, these lesions can be macroscopically confused with nasal polyposis. Pain is a late symptom, which can be associated with increasing facial bulging, being considered, in these cases, as a sign of tumor infiltration; however, the symptoms vary depending on the location of the disease. Impaired vision and headaches may also be present, but less frequent.^4^, ^6^, ^9^

The differential diagnosis of SNHPC includes juvenile hemangioma, glomus tumor, angiosarcoma, leiomyoma, leiomyosarcoma, schwannoma, mesothelioma, liposarcoma, benign or malignant histiocytoma, solitary fibrous tumor, synovial sarcoma, chondrosarcoma, neuroblastoma, and adenoid cystic carcinoma.^1^, ^4^, ^12^

A challenging situation that deserves our attention during diagnostic investigation is about the possible cases of TIO. The diagnosis and treatment are usually late because the illness symptoms (hypophosphatemia, progressive bone pain, muscle weakness, walking difficulties and other symptoms) are nonspecific in the initial phase, and the histological diagnosis is confounded with other diseases (phosphaturic mesenchymal tumor, giant cell tumor, osteosarcoma, hemangioma, HPC, and others). The cases of TIO caused by SNHPC are extremely rare, representing less than 0.5% of all cases and about 5% from HPCs from other sites. However, it is advisable to remember this differential diagnosis in cases of osteomalacia of unknown cause in patients with nasal polypoid masses.^10^

The preoperative evaluation requires detailed evaluation of imaging tests (CT and NMR).^3^ In the case of large tumors and because it is a hypervascularized tumor with a branched vascular network of different calibers, the best form of evaluation is with an angiography, which will also assist in the planning of preoperative embolization. Biopsy of the lesion is not recommended at first due to the high risk of profuse bleeding.^1^, ^9^

Although embolization presents risks—ischemia of other organs, facial flushing, thrombotic complications during catheter implantation—these are less likely than the massive hemorrhage that can occur during surgery, especially if performed with a meticulous angiographic technique, taking care of the possibility of reflux of thrombolytic agents to unwanted areas. The angiographic study of a hypervascularized tumor must be considered, even without the previous diagnosis, for the performance of preoperative embolization, as it provides more safety in the surgery and less blood loss in the procedure.^1^, ^9^, ^13^

Diagnostic confirmation of SNHPC is performed by anatomopathological examination, complemented by immunohistochemistry. Before the routine use of such exams, the diagnosis was commonly confused with other types of tumors, due to the similar vascularization that these diseases present, which are the blood vessels in the shape of “deer's antler”.^4^, ^6^, ^9^, ^12^ In the immunohistochemistry study, vimentin and CD34 are considered as markers reliably detected in SNHPC tumor cells.^6^, ^9^, ^14^

The therapy of choice for SNHPC is undoubtedly broad surgical resection with safety margins. Neck dissection is not indicated because cervical metastasis rarely occurs in the HPC. Although there appears to be no difference in the therapeutic outcome between endoscopic resection and conventional open surgery (lateral rhinotomy), the endonasal endoscopic approach offers some advantages compared to the external approach. Among them, the endonasal approach shows a wide and magnified view of the entire lesion and the structures of the rhinopharynx, helping to accurately assess the insertion of the SNHPC, as well as the margins and surrounding tissues. Other benefits include preservation of nose physiology and reduced risk of damaging the lacrimal apparatus.^1^, ^5^, ^13^, ^15^, ^16^ As mentioned above, the endoscopic and embolization approach in SNHPC are already well discussed in the literature, but there is no case report in the literature of SNHPC in a Jehovah's Witness patient, much less the discussion of the challenges in the management that this situation imposes. The main treatment modalities described for nasosinusal HPC are given in Table 1.

**TABLE 1:** Summary of the main treatment modalities described for sinonasal hemangiopericytoma

Modality	Comments
Open resection	Primary treatment used in most previously published articles. Increased morbidity and presence of facial scars.
Endoscopic resection	Less morbidity and absence of external scars. No difference in recurrence rates compared to open access in both complete and incomplete resections. Currently it is the preferred primary treatment modality, when possible.
Embolization	Used preoperatively aiming to reduce risks of intraoperative bleeding and facilitate complete resection. Possibility of important side effects depending on the embolized vessel.
Chemotherapy	Only few studies had described de use of chemotherapy as primary treatment modality for SNHPC. No evidence for or against the use.
Radiotherapy	High rates of recurrence and tumor‐related death when used as primary treatment modality. Used as an adjunct therapy when there is incomplete surgical resection.

The size and infiltration of the tumor are prognostic factors that influence the survival of patients with SNHPC. This fact is aggravated in cases of extensive tumors located on the head and neck because, due to the complexity of their anatomical structures, it is rarely possible to perform a radical resection with safety margins. Even with all these adversities, the importance of disease‐free surgical margins is recommended in the treatment of HPC. Another prognostic factor is the histological degree of the lesion; mitotic rare is the most frequently cited pathological feature to distinguish benign or low‐grade tumors from the malignant or high‐grade ones. In comparison with the well and/or moderately differentiated HPC, the poorly differentiated presents with more than four mitoses per microscopic field, being an unfavorable prognostic factor for the patient's survival.^5^, ^16^

Wushou et al. in his metanalysis of 116 head and neck's HPC cases mention, among the main findings, that surgery is the first treatment choice, resulting in a good prognosis, while the therapeutic results of surgery associated with adjuvant radiotherapy were not superior to surgery alone. For other authors, the decision to add radiotherapy to surgery can increase disease‐free survival and decrease the rate of recurrence, especially in cases of extensive and deep HPC in which complete resection was not possible, or which have poorly differentiated tumors, or the ones who were primarily operated with partial tumor resection or patients with compromised margins.^1^, ^5^, ^16^, ^17^, ^18^

Chemoradiotherapy as an exclusive treatment has limited efficacy and should be reserved as a palliative treatment for cases whose surgical resection presents a very high risk of death or in cases of advanced metastases.^6^, ^14^ Although there are several articles that used adjuvant chemotherapy or chemoradiotherapy applied in the treatment of head and neck HPC, the limited number of cases and follow‐up prevents a direct comparison of these results with other forms of treatment instituted.^5^

Tanigawa et al. described a case of SNHPC successfully treated with recombinant interleukin 2 (rIL‐2). It is believed that rIL‐2 attacks directly the tumor cells, as well as activates natural killer cells (NK cells), triggering anti‐tumor effects. In this case, after administration of rIL‐2, increased activity of NK cells and reduction of tumor size were also observed. Thus, a broad external approach (lateral rhinotomy) could be performed, after previous selective embolization of the tumor‐nourishing artery to obtain a good visualization and en‐bloc resection of the entire lesion, showing it to be an alternative for the treatment of SNHPC.^15^

The HPC located in the nasal region is often characterized by a benign nature, with a low tendency to metastasize. However, even in the context of a surgical resection of the tumor, whether through the endoscopic or open route, the SNHPC local recurrence rate remains high, around 26.7%, with an overall survival in 2 and 5 years of 93% and 86%, respectively.^2^, ^4^, ^5^, ^9^, ^16^

The HPC prognosis is still not predictable, neither by the clinical aspect, nor by histological findings. Local recurrence can occur after a prolonged disease‐free interval, with an average of 46 months after surgical resection, with cases reported in the literature that relapses 26 years after the initial surgery. Depending on the size of the SNHPC, its infiltrative nature and time of evolution, a new surgery is considered the standard treatment. Recurrence, when present after a primary resection, indicates a poor prognosis and often precedes the development of distant metastases, which in certain cases, may be the only way to find out about the malignant nature of SNHPC.^1^, ^2^, ^4^, ^9^, ^16^

Distant metastasis of head and neck HPC is infrequent, appears late and can, with some frequency, affect the brain, lung, mediastinum, chest wall, bones, abdomen and liver; a rare case of thyroid metastasis is reported in the literature.^1^, ^5^, ^6^, ^14^

The SNHPC should be better assessed preoperatively as a tumor with malignant potential than a properly benign one, due to its possible capacity for more aggressive behavior and distant metastases. Thus, strict and frequent surveillance throughout the life of patients with HPC should be mandatory.^16^

## 3 CONCLUSION

We report here a rare case of an extent sinonasal HPC of a female patient who, in addition to her limiting clinical conditions, had restrictions on blood components by religious belief. In order for the concept of bloodless surgery to be applied, a multidisciplinary effort was required, which included, in addition to all the strategies and techniques available to prevent blood transfusion, the inclusion and commitment of the entire team, from the preoperative evaluation of the patient to the day of her hospital discharge. In this case report, endoscopic tumor surgery linked to preoperative embolization proved to be an excellent treatment method for complete resection of SNHPC, providing us with conditions for better hemostasis, which is essential to avoid large bleedings and risk of blood transfusion, mainly in this select group of patients. This appears to be the first case report of SNHPC in a Jehovah's Witness patient as well as the first to discuss the challenges inherent in managing this situation.

## CONFLICT OF INTEREST

The authors declare no conflicts of interest.

## ETHIS STATEMENT

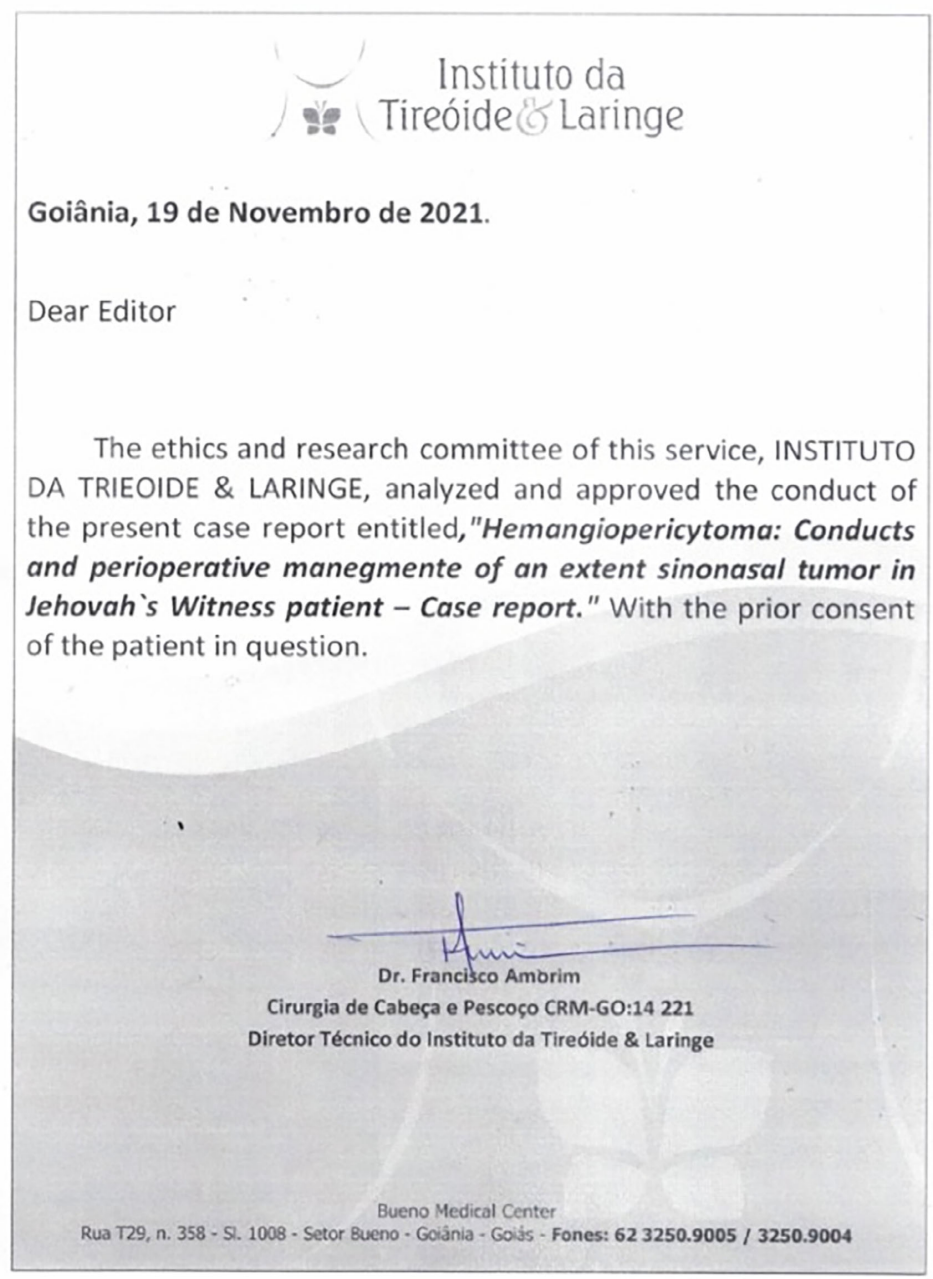

## AUTHOR CONTRIBUTIONS

**Francisco de Souza Amorim Filho:** Conceptualization (equal); supervision (equal); writing – original draft (equal); writing – review and editing (equal). **Flávio Mignone Gripp:** Supervision (equal); writing – review and editing (equal). **Guilherme Souza de Faria:** Writing – original draft (equal). **Mateus Capuzzo Gonçalves:** Writing – original draft (equal). **Lincoln Miyahira:** Conceptualization (equal); supervision (equal); writing – original draft (equal); writing – review and editing (equal).

## Data Availability

The data that support the findings of this study are available from the corresponding author upon reasonable request.

## References

[1] Fareed MM , Al Amro AS , Akasha R , et al. Parapharyngeal space hemangiopericytoma treated with surgery and postoperative radiation–a case report. Head Neck Oncol. 2012;4:10. 22480217 10.1186/1758-3284-4-10 PMC3349527

[2] Auguste LJ , Razack MS , Sako K . Hemangiopericytoma. J Surg Oncol. 1982;20:260‐264. 7109630 10.1002/jso.2930200416

[3] Stout AP , Murray MR . Hemangiopericytoma: a vascular tumor featuring Zimmermann's pericytes. Ann Surg. 1942;116:26‐33. 17858068 10.1097/00000658-194207000-00004 PMC1543753

[4] Espat NJ , Lewis JJ , Leung D , et al. Conventional hemangiopericytoma: modern analysis of outcome. Cancer. 2002;95:1746‐1751. 12365023 10.1002/cncr.10867

[5] Wushou A , Miao XC , Shao ZM . Treatment outcome and prognostic factors of head and neck hemangiopericytoma: meta‐analysis. Head Neck. 2015;37:1685‐1690. 24954602 10.1002/hed.23812

[6] Camacho LHG , León AJ , Paulo FP , Ramírez RP , Sala CJJ , Ortiz TP . Hemangiopericitoma. Nasal Rev Mex. 2013;2:93‐97.

[7] Proietti A , Sartori C , Torregrossa L , et al. A case of metastatic haemangiopericytoma to the thyroid gland: case report and literature review. Oncol Lett. 2012;3:1255‐1258. 22783428 10.3892/ol.2012.661 PMC3392569

[8] Navarrete ML , Maeso J , Pellicer M . Hemangiopericytoma of the nasal septum. Eur Arch Otorhinolaryngol. 1990;247:384‐386. 2278706 10.1007/BF00179014

[9] Ledderose GJ , Gellrich D , Holtmannspötter M , Leunig A . Endoscopic resection of sinonasal hemangiopericytoma following preoperative embolisation: a case report and literature review. Case Rep Otolaryngol. 2013;2013:796713. 23738175 10.1155/2013/796713 PMC3659647

[10] Li J , Huang Y , Yang F , Zhang Q , Chen D , Wang Q . Sinonasal hemangiopericytoma caused hypophosphatemic osteomalacia: a case report. Medicine (Baltimore). 2018;97:e13849. 30593185 10.1097/MD.0000000000013849 PMC6314754

[11] Adelola OA , Ahmed I , Fenton JE . Management of Jehovah's witnesses in otolaryngology, head and neck surgery. Am J Otolaryngol. 2008;29:270‐278. 18598840 10.1016/j.amjoto.2007.08.006

[12] Enzinger FM , Smith BH . Hemangiopericytoma. An analysis of 106 cases. Hum Pathol. 1976;7:61‐82. 1244311 10.1016/s0046-8177(76)80006-8

[13] Morandi U , Stefani A , De Santis M , Paci M , Lodi R . Preoperative embolization in surgical treatment of mediastinal hemangiopericytoma. Ann Thorac Surg. 2000;69:937‐939. 10750792 10.1016/s0003-4975(99)01361-2

[14] Zanasi Junior S , Lozano PAM , Sá VHLC , Pereira Filho GV , Heinke T . Hemangiopericitoma de órbita. Rev Bras Cir Plást. 2012;27:487‐489.

[15] Tanigawa T , Tanaka H , Kano F , Ueda H , Inafuku S . Nasal hemangiopericytoma successfully treated with a combination of rIL‐2 and extranasal approaches. J Surg Case Rep. 2017;2017:rjx202. 29423144 10.1093/jscr/rjx202 PMC5798123

[16] Duval M , Hwang E , Kilty SJ . Systematic review of treatment and prognosis of sinonasal hemangiopericytoma. Head Neck. 2013;35:1205‐1210. 22733718 10.1002/hed.23074

[17] Krengli M , Cena T , Zilli T , et al. Radiotherapy in the treatment of extracranial hemangiopericytoma/solitary fibrous tumor: study from the rare cancer network. Radiother Oncol. 2020;144:114‐120. 31805515 10.1016/j.radonc.2019.11.011

[18] Koscielny S , Bräuer B , Förster G . Hemangiopericytoma: a rare head and neck tumor. Eur Arch Otorhinolaryngol. 2003;260:450‐453. 12759763 10.1007/s00405-003-0625-8

